# Simplex-lattice design and decision tree optimization of endophytic *Trichoderma*-multi-walled carbon nanotube composite for enhanced methylene blue removal

**DOI:** 10.1016/j.heliyon.2024.e39949

**Published:** 2024-10-29

**Authors:** Sahar E. Abo-Neima, Emad M. Elsehly, Fatimah O. Al-Otibi, Mohammed M. El-Metwally, Yosra A. Helmy, Noha M. Eldadamony, WesamEldin I.A. Saber, Adel A. El-Morsi

**Affiliations:** a Physics Department, Faculty of Science, Damanhour University, Damanhour, 22511, Egypt; b Botany and Microbiology Department, Faculty of Science, King Saud University, Riyadh, 11451, Saudi Arabia; c Botany and Microbiology Department, Faculty of Science, Damanhour University, Damanhour, 22511, Egypt; d Department of Veterinary Science, Martin-Gatton College of Agriculture, Food, and Environment, University of Kentucky, Lexington, KY, 40546, USA; e Seed Pathology Department, Plant Pathology Research Institute, Agricultural Research Center, Giza, 12619, Egypt; f Microbial Activity Unit, Microbiology Department, Soils, Water and Environment Research Institute, Agricultural Research Center, Giza, 12619, Egypt; g Botany Department, Faculty of Science, Mansoura University, Mansoura, 35516, Egypt

**Keywords:** Dye reduction, Carbon nanotubes, Experimental design, Functionalization, SEM, FT-IR

## Abstract

This study investigates a novel approach for enhancing methylene blue (MB) removal from water using a composite of endophytic *Trichoderma* mate and multi-walled carbon nanotubes (MWCNTs). For the first time, a unique combination of simplex-lattice design and decision tree learning algorithm was employed to optimize MB removal. This innovative approach effectively identified the optimal composite ratio of hyphal mate (0.5354 g/L) and MWCNTs (0.4646 g/L) for maximizing MB removal, which achieved remarkable removal efficiency ranging from 63.50 to 95.78 % depending on the combination used. The DT model further demonstrated promising potential for predicting MB removal efficiency. SEM revealed a unique hybrid material formed by the intertwining or entrapment of MWCNTs within the hyphal network of Trichoderma mate. FT-IR analysis confirmed the presence of novel functional groups on the MWCNTs' surface at 2438.79 and 528.25 cm^−1^, likely due to interactions with the endophytic fungi's biomolecules. These functional groups presumably act as reducing and stabilizing agents, promoting efficient MB adsorption. This research paves the way for utilizing the combined biological and chemical approach (fungal biomass and MWCNTs) in bioremediation applications. The findings suggest significant potential for practical applications in wastewater treatment, providing an eco-friendly and cost-effective method for dye removal. Furthermore, the proposed method shows promise for scaling up to industrial wastewater treatment and applicability in resource-limited settings, offering a sustainable solution for global water pollution challenges. Further investigations with larger datasets incorporating additional influencing factors are necessary to refine the predictive power of the DT model for practical applications.

## Highlights

• Novel *Trichoderma*-MWCNTs composite for MB removal.
• First use of simplex-lattice design and decision tree for maximizing MB removal.
• Unique hybrid material with a novel composite structure.
• New functional groups on MWCNTs.

## 1 Introduction

In the textile industry, the dyeing process is crucial, with optimization of dyeing techniques being essential to minimize dye loss, improve product quality, reduce costs, and mitigate environmental impact. The severity of dye pollution is evident, with unbounded dye ranging from 1 to 50 % depending on the dye type [1]. Azo dyes, comprising 60–70 % of the total used dyes due to their stability, color variations, and low prices, are particularly prevalent. Concerningly, some dyes, including methylene blue, exhibit acute toxicity and potential carcinogenic and mutagenic effects, posing immediate and long-term risks to organisms [2]. This issue scales to a global challenge, with an estimated 280,000 tons of textile dyes annually discharged in industrial effluents worldwide [3]. Recent epidemiological studies have correlated long-term exposure to dye-contaminated water with increased incidences of certain cancers, reproductive disorders, and developmental issues [4]. Furthermore, bioaccumulation of these dyes in aquatic organisms can lead to their entry into the human food chain, exacerbating health risks [5]. Considering these global challenges, our study on methylene blue removal using a novel composite material gains additional significance, contributing to broader efforts in mitigating the environmental, economic, and health impacts of textile dye pollution.

Methylene blue (MB), a versatile dye extensively used for coloring fabrics like silk, wool, and cotton, contributes significantly to textile dye pollution in wastewater. Its toxicity poses threats to aquatic life, potentially causing harmful effects. Regulations are being implemented to limit MB use, and researchers are exploring eco-friendly alternatives for a sustainable textile industry. Developing effective and eco-friendly methods to remove MB from wastewater is therefore a pressing need. Among the various methods explored for MB removal, biological treatment using microorganisms, and their composites has gained attention in recent years due to their cost-effectiveness, sustainability, and versatility [6,7].

Confined to the nanoscale, materials exhibit dramatic transformations in their thermal, optical, and mechanical properties compared to their bulk counterparts. For example, nanoparticles often demonstrate enhanced heat conductivity, tunable light absorption, and exceptional strength-to-weight ratios [8]. Carbon nanotubes (CNTs), are a promising nanomaterial for enhancing the removal of methylene blue from wastewater [9]. CNTs are cylindrical structures composed of carbon atoms, with a high surface area, unique electronic and mechanical properties, and excellent adsorption capabilities. They have been extensively studied for various environmental applications, including water treatment, due to their high reactivity and cc activity [9,10]. CNTs stand out as groundbreaking nanostructures showcasing a remarkable suite of physical properties. Their diminutive size, often thousands of times thinner than human hair, combined with their exceptionally high aspect ratio, unlocks a fascinating spectrum of electrical, mechanical, and thermal properties, unlike any bulk material [11].

Multi-walled carbon nanotubes (MWCNTs), comprising concentric graphene sheets, have emerged as a revolutionary material due to their unique features like high strength, conductivity, and surface area. Beyond applications in sensors, filtration, and lightweight composites [12,13], MWCNTs hold promise as multifunctional nanotools for drug delivery, tissue engineering, and targeted therapies [14]. Their high surface area, chemical stability, and strong adsorption capabilities enable capturing pollutants like methylene blue (MB). The unique structure of MWCNTs facilitates interaction with dye molecules through π-π interfaces, electrostatic attractions, and van der Waals forces, leading to enhanced adsorption efficiency. Their potential as multifunctional nanotools is even more exciting, with researchers exploring their use in drug delivery, tissue engineering, and targeted therapies. Recent advancements in MWCNT-based biocompatible structures, for example, hold promise for regenerative medicine and targeted cancer treatment [14,15].

*Trichoderma* spp., particularly endophytic strains, represent a genus with remarkable multi-biological functionalities. These endophytes, constituting a significant portion of the plant microbiome, exhibit diverse bioactivities with applications in various fields [16]. Notably, *Trichoderma* spp. outshine in biodegradation and biosorption due to their robust enzyme systems and biomass composition. They produce many extracellular enzymes and metabolites capable of breaking down complex organic pollutants, including synthetic dyes. Additionally, the extensive network of fungal hyphae offers a large surface area enriched with functional groups that effectively bind to dye molecules, further enhancing overall removal efficiency [[17], [18], [19]].

Traditional dye removal methods, such as activated carbon adsorption and chemical precipitation, often face limitations in terms of efficiency and cost-effectiveness [20,21]. Our approach leverages the high adsorption capacities and stability of MWCNTs combined with the biological degradation and additional binding sites provided by Trichoderma sp. This synergistic effect significantly enhances MB removal efficiency, offering a more sustainable and economical solution than the traditional approaches [22].

To optimize the conditions for MB reduction by a composite made of *Trichoderma* sp. and MWCNTs. The simplex-lattice design was employed as a powerful statistical tool that enables the exploration of multiple variables and their interactions and can potentially reveal new insights into the interactions between the causal factors and response variable(s) and statistically determine the optimal combination. Accordingly, the simplex design can find the best solution to a problem by iteratively adjusting a set of parameters and evaluating the results [[23], [24], [25]].

This study employed decision trees (DTs), a supervised machine learning technique within the realm of data mining algorithms. DTs excel at various tasks, including classification, regression, and uncovering the relative significance of independent variables on a response target. Furthermore, they provide useful tools for analyzing and interpreting data from various sources. DTs function by iteratively partitioning a dataset into progressively smaller subsets until each subset achieves homogeneity concerning the target variable. The training data guides the learning process, establishing decision rules at each node within the tree structure. These decision rules then empower the model to predict the target variable for previously unseen data points [26,27]. To ensure a DT makes accurate predictions on new data, it requires a diverse and representative dataset. Rigorous validation is crucial to prevent overfitting and bias, employing various techniques, such as cross-validation and regularization, can help mitigate these issues and enhance the model's generalizability [26]. Based on the existing literature, simplex design and DT methodologies have not been previously employed for modeling MB removal. This innovative approach has the potential to offer a fresh perspective on the dye removal process by integrating both statistical and machine learning techniques.

The simplex-lattice design was chosen for its effectiveness in optimizing component combinations within mixture experiments [23,24]. DT was employed for their ability to classify and determine variable significance [26,27]. The sample size was determined based on model complexity and statistical analysis needs [28]. While sufficient for current validity, enough datasets could potentially enhance the DT model's predictive power.

Based on our current understanding, there is currently no literature on the utilization of endophytic fungi i.e., *Trichoderma* sp., as a biological system in conjunction with MWCNTs for the bioremediation of MB. Furthermore, no prior studies have incorporated the simplex-lattice design or DT in similar investigations. As a response to this gap, we employed a combination of *Trichoderma* sp. and MWCNTs to assess the potential of this composite in the bioremediation of MB dye. Our study is specifically designed to explore the efficacy of a composite comprising *Trichoderma* sp. hyphal mate and MWCNTs in the removal of MB from water.

## 2 Materials and methods

**Fig. 1:**
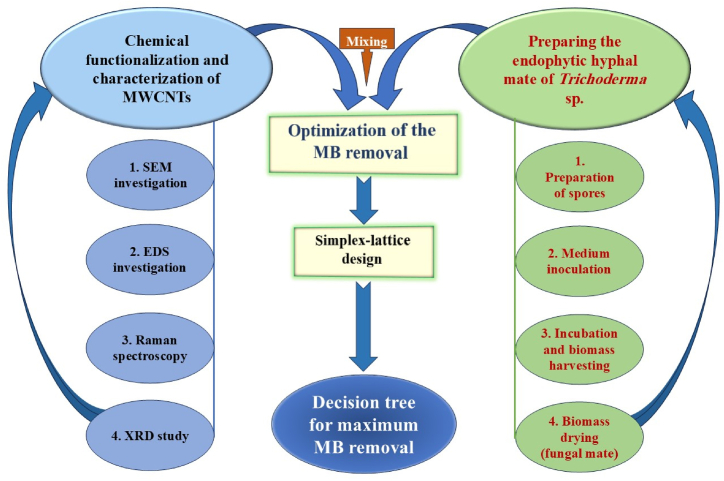
A schematic diagram of the experimental setup showcases the overall stages of the preparation of the materials (*Trichoderma* sp. mate and multi-walled carbon nanotubes) up to the design experiments for methylene blue removal.

Figure (1) illustrates the sequential steps involved in the optimization of MB removal using a composite material composed of chemically functionalized MWCNTs and endophytic hyphal mate of *Trichoderma* sp. The diagram outlines the experimental workflow, starting with the characterization of the functionalized MWCNTs and the preparation of fungal spores. Subsequently, the simplex-lattice design was employed to optimize the combination of MWCNTs and fungal mate for maximum MB removal efficiency. The DT paradigm was then utilized to model and predict the optimal conditions for MB removal.

### 2.1 Preparation of MB

The cationic dye methylene blue, analytical grade, was obtained from Sigma-Aldrich, Cairo office, Egypt, and used as a pollutant model. A standard stock solution of MB (100 mg/L) was prepared by dissolving 0.01 g of the dye powder in 100 mL of distilled water. The solution was stored in a dark, sealed container at 4 °C for further use.

### 2.2 Preparing the hyphal mate of *Trichoderma* sp.

The used fungal model: *Trichoderma* sp. NW12 was previously isolated in the Microbial Activity Unit, Microbiology Department, Soils, Water and Environment Research Institute, Agricultural Research Center (ARC), Giza, Egypt. According to the provider, *Trichoderma* sp. was isolated as an endophytic fungus from common bean plants. In a typical process, spore suspension (10^7^ per ml) of the fungus was prepared from a 7-day-old culture grown on plates of potato dextrose agar medium. This inoculum was used to inject 100 mL potato dextrose broth medium contained in 500 ml Erlenmeyer flasks at the rate of 10 %. The inoculated flasks were incubated at 28 °C ± 2.0 on an orbital shaker at 150 rpm for 5 days. After growth, the fungal biomass was separated by filtration using a sterile nylon cloth. The biomass was then washed thoroughly with deionized water to remove the residues. The hyphal mate was dried at 50 °C to constant weight before use.

### 2.3 Preparation of MWCNTs

The current material used is multi-walled carbon nanotubes (Taunit-MD) in powder form, which was delivered by the Nanotech Centre in Tambov, Russia with a purity of 99.5 %. MWCNT specifications were free, dark powder made up of granular agglomerated nanotubes with small diameters and short lengths. The mathematical parameters and some physical properties were ≥3 μm in length, 28–39 nm of outer diameter, and 6–8 nm of inner diameter, with a specific surface area of 180–220 m^2^/g, and bulk density of 0.05–0.06 g/cm^3^.

#### 2.3.1 Chemical functionalization

The surface functionalization of MWCNTs was performed to improve the dispersibility, increase the adsorption capacity, and improve the stability of MWCNTs in water by attaching functional groups. Two steps were applied to purify and functionalize the MWCNTs: the first involved purifying the pristine sample to remove amorphous carbon and metal catalysts with HCl and H_2_O_2_ mixture. In a 150 ml open flask, 100 mg of pristine MWCNTs were combined immediately with 20 ml of 5 mol L− 1 hydrochloric acid and 20 ml of 50 % H_2_O_2_. The entire mixture was then subjected to ultrasonic vibrations for 2 h at 60 °C in a water bath. Each time the mixture was filtered after 30 min, 20 mL of hydrochloric acid and 20 mL of H_2_O_2_ were added to the mix once again [29], The second step involved oxidizing the purified MWCNTs with nitric acid. The same amount of pristine MWCNTs was dispersed in 100 ml of nitric acid 60 % in a 250 ml flask, using the ultrasonic technique. The mixture was then sonicated for 8 h at 80 °C in a water bath. To achieve a pH level of 7, the mixture was separated and washed several times with deionized water. The acquired MWCNT samples were dried in the furnace at 80 °C for 12h [30].

#### 2.3.2 MWCNTs characterization

The surface morphology and elemental composition of the MWCNTs were explored using scanning electron microscopy (SEM, FESEM, Quattro S, Thermo Scientific) equipped with energy-dispersive X-ray spectroscopy (EDS) at a resolution voltage of 20 KeV. Before analysis, the sample was dried and sputter-coated with gold. A powerful technique to investigate the crystallinity of the tubes in the samples, micro-Raman spectroscopy (laser wavelength 632.8 nm) was employed to probe for defects in the MWCNT structure, in back-scattering mode. X-ray diffraction (XRD, Difray 401, Scientific Instruments, Russia) using Cr-Kα radiation (λ = 2.2909 Å) was performed at room temperature to inspect the crystalline structure before and after functionalization [31].

### 2.4 Theory, and calculation of simplex-lattice design (SLD)

The influence of two causal factors (hyphal mate, and MWCNTs) on the removal of MB dye was investigated using the SLD mixture with a lattice degree of 2. The design is normally applied for systems involving compositions, in which the sum of the proportions must be unity (100 %) as in our case study here. The optimal combination of hyphal mate and MWCNTs that maximize the removal of MB was investigated in 100 ml working volume, containing 100 mg/L MB. The range of each of the two causal factors (hyphal mate, and MWCNTs) ranged between 0 and 1 g/L. The select reference point was at 0.5 g/L for each of the causal variables. The hyphal mate and MWCNTs concentrations in the composite system were simultaneously optimized using the SLD with a 0.25 g/L step size, maintaining their sum at 100 %. Accordingly, the experimental design is shown in Table (1).

**Table 1:** The array of simplex-lattice mixture experimental design of the two predictors (hyphal mate, and multi-walled carbon nanotubes) for methylene blue removal rate and the predicted and fitted values, as well as terminal nodes of the decision tree.

Formulation	Causative factors (g/L)		Methylene blue removal, %					
			Actual	SLD		DT		
	Hyphal mate (g/L)	MWCNTs (g/L)		Fitted	Error	Fitted	Error	Terminal node
1	0.50	0.50	95.78 ± 1.20	93.39	2.39	94.89	0.90	3
2	0.25	0.75	85.01 ± 0.08	85.16	−0.15	84.03	0.98	2
3	0.25	0.75	83.04 ± 1.06	85.16	−2.12	84.03	−0.98	2
4	0.00	1.00	63.50 ± 0.29	64.08	−0.58	64.40	−0.90	1
5	1.00	0.00	70.86 ± 0.20	71.27	−0.41	71.45	−0.59	5
6	0.50	0.50	93.99 ± 0.30	93.39	0.6	94.89	−0.89	3
7	0.75	0.25	89.07 ± 0.16	88.76	0.31	87.90	1.18	4
8	0.75	0.25	86.72 ± 1.02	88.76	−2.04	87.90	−1.18	4
9	1.00	0.00	72.04 ± 0.39	71.27	0.77	71.45	0.59	5
10	0.00	1.00	65.30 ± 0.61	64.08	1.22	64.40	0.90	1

Flasks containing the reaction mixture were kept at room temperature for 60 min, on an orbital shaker at 50 rpm. Upon experimenting, all runs were centrifuged at 10000 rpm for 20 min to remove the absorbent (hyphal mate and MWCNTs composite). The residual MB was determined UV–Vis spectrophotometrically at 668 nm, at each point of the simplex. The reduction percentage in MB was calculated concerning the initial concentration and expressed as the removal efficiency % of MB (Equation (1)).

(Equation 1) Removalefficiency%=100×(C1−C2)/C1

Where C1 represents the initial concentration of methylene blue, while C2 denotes the residual concentration after treatment.

The rigorous statistical analysis of the SLD mixture results pinpointed the optimal combination of hyphal mate and MWCNT concentrations for achieving maximum MB removal. The arithmetical association between the levels of the input factors and the response variable based on experimental data can be used to optimize the input factors to achieve the desired response. The following empirical model (Equation (2)) was used to calculate the relationship between the causative and response factors:

Equation (2) Y=β1X1+β2X2+β12X1X2

Where Y is the MB removal percentage, X_1_ and X_*2*_ are the process variable hyphal mate, and MWCNTs, respectively. β_1_ and β_1_ are the coefficients of regression for hyphal mate, and MWCNTs, respectively. β_12_ is the mutual coefficient.

The ANOVA was performed to identify the significant interactions between hyphal mate and MWCNT concentrations. Response surfaces of MB removal were visualized to understand their impact on the response variable. Based on multiple criteria including correlation coefficient (R^2^), adjusted R^2^, predicted R^2^, and (the predicted residual sum of squares) PRESS, the model with the best fit was identified. This model predicted the optimal formulation for maximizing MB removal. The predicted values were then compared to experimental results to validate the accuracy of the response surface.

### 2.5 Learning algorithm of DT

A parametric machine learning regression tree was constructed to model the maximization of MB sorption with the aid of the *Trichoderma* sp. mate and the functionalized MWCNTs composite (independent variables) that were used during the SLD. The two predictors (*Trichoderma* sp. mate and MWCNTs) were used to train the DT. This study evaluated the relative influence of experimental design factors (predictors) on the reduction of noise introduced by the procedures. The analysis focused on their impact on achieving the desired outcome (maximum MB removal). The DT approach was employed, utilizing continuous data points for node splitting based on the least squares error criterion. The maximum correlation coefficient (R^2^) was considered for selecting the optimum DT. The minimal number of cases to split both the internal node, and that allowed for the terminal node was evaluated.

To select and evaluate the best decision tree model, various performance metrics were analyzed on both the training and testing datasets. These metrics included the coefficient of determination (R^2^) and root mean squared error (RMSE), mean squared error (MSE), mean absolute deviation (MAD), mean absolute percentage error (MAPE), and standard deviation. For modeling maximum MB removal, the validation method using several numbers of folds (K) was tested based on K-fold cross-validation for evaluating the DT model performance. In which, the model is trained K times, each time using K-1 folds and the remaining fold as testing data. This means that in each iteration, a different fold is used as the testing set, while the rest are used for training.

To assess the accuracy of the predictions and prevent overfitting, 10 data points obtained from two repetitions of the SLD experiment were employed. During the training of DT, the nodes were continuously divided until they reached a point where further division wouldn't improve the model. While deeper trees can result in more precise predictions, the tree's growth was halted when further modifications did not reduce the MSE.

To validate the DT model experimentally, experiments were conducted based on the conditions predicted at various nodes of the DT for different mixtures of the investigated predictors. The predicted values were then compared to the observed values to assess the model's effectiveness in maximizing MB removal.

### 2.6 SEM investigation

The surface structure of MWCNTs was investigated using scanning electron microscopy (SEM) at 10 kV resolution. Both MWCNTs and Trichoderma sp. were fixed with 2.5 % glutaraldehyde (4 °C, 12 h), dehydrated in ascending ethanol grades (25–100 %), dried, and sputter-coated with gold for SEM analysis with SEM, Model: JSM-IT200 JEOL Ltd., Tokyo, Japan [32].

### 2.7 Fourier transform infrared (FT-IR)

A small disc, prepared by mixing 5 mg of the sample with 100 mg of KBr, was analyzed using FT-IR spectroscopy (Vertex 70 RAM II IR Bruker spectrometer, Germany) to identify potential active groups in biomolecules associated with nanoparticle capping and reduction. The FT-IR spectra of prepared samples were acquired within the range of 450–4500 cm⁻^1^. The data was plotted with transmittance (%) on the y-axis and wavenumber (cm⁻^1^) on the x-axis.

### 2.8 Software and statistical procedures

The experiments were conducted twice, each of three biological replicates, and the results were averaged ± standard deviation. The SLD mixture and learning algorithm of DT were generated and analyzed using Minitab software (version 21, Minitab Inc., State College, PA, USA). The data generated from the SLD experiment was employed as training data for a machine-learning model of DT.

## 3 Results and discussion

### 3.1 Surface structure of *Trichoderma* sp. NW12 mate

**Fig. 2:**
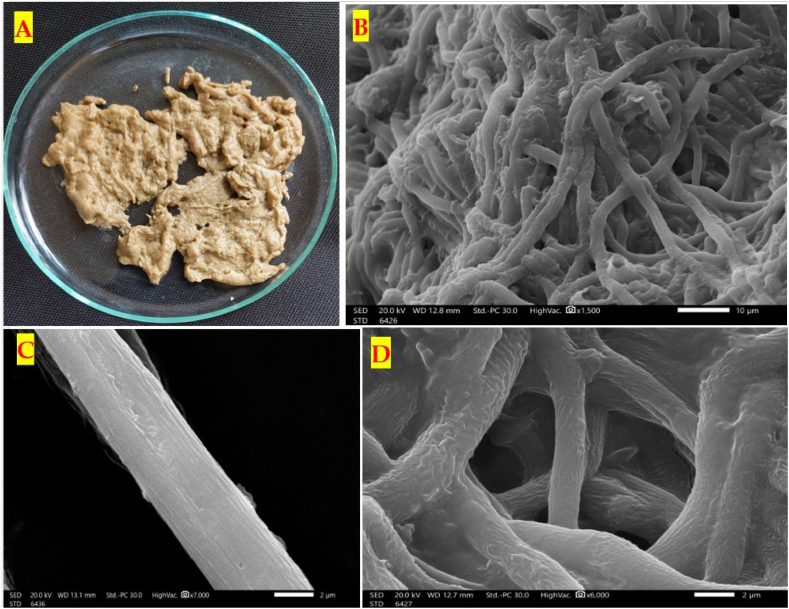
Zoom-in view of the fresh *Trichoderma* mate in Petri plate (A), and the images of scanning electron microscope of the surface topology of the normal hyphal mate (B, C & D) at various magnifications.

The image of the prepared hyphal mate of the endophytic *Trichoderma* sp. is introduced in Figure (2). The freshly prepared hyphae of *Trichoderma* sp. on the Petri plate are creamy in color and have a bulk structure (Fig. 2 A). The detailed and magnified view of the intricate and complex structure of *Trichoderma* mate at a microscopic level showcases the mate appears as a network of branching hyphae (Fig. 2 B, C, and D), which are the thread-like structures that make up the mycelium of the fungus. The hyphae are typically slender, elongated, and interconnected, forming a dense and web-like structure. The surface hypha is smooth to slightly rough to allow efficient movement and colonization through the surrounding environment and on different substrates. The slight roughness here may have occurred during the preparation of the dried hyphal mate.

### 3.2 Characterization of the functionalized MWCNTs using SEM and EDS

#### 3.2.1 SEM investigation

**Fig. 3:**
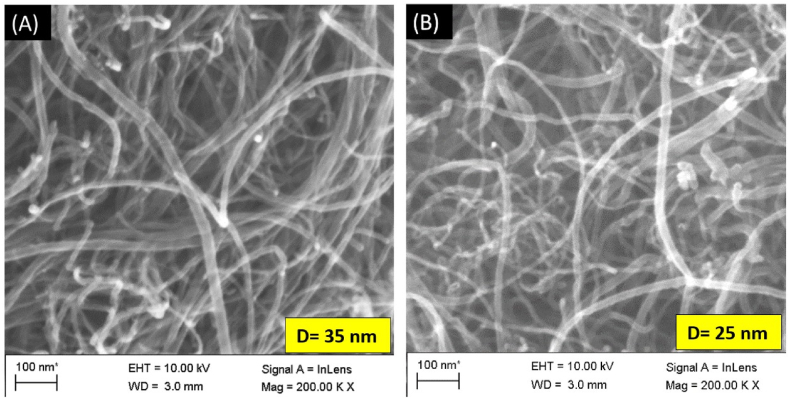
The scanning electron microscope micrograph, illustrating the surface morphology and the average tube diameter (D) of raw (A), and after (B) functionalization of multi-walled carbon nanotubes.

The micrograph of SEM was used to describe the structural and morphological features of pristine and oxidized MWCNTs. The reduced multi-walled carbon nanotubes (R-MWCNTs) (Fig. 3 A) appear to be more crystalline in structure than the oxidized multi-walled carbon nanotubes (O-MWCNTs) (Fig. 3 B). The O-MWCNTs diameter and tube length were lower than the pristine R-MWCNTs. The lower tube diameter with increased surface area may be because of oxidation during the functionalization process. These parameters are expected to influence the MWCNTs' performance [30,33].

Functionalization of MWCNTs introduces chemical groups onto their surfaces. These groups disrupt the strong van der Waals interactions between nanotubes, promoting better dispersion or complete solubilization in surrounding liquids or solids [34]. As a result, functionalized MWCNTs become more reactive, allowing for further chemical modifications like metal attachment, grafting reactions, and ion adsorption. The functional groups also act as anchoring sites, facilitating the bonding of other molecules and enabling further chemical tailoring [15].

#### 3.2.2 EDS investigation

**Fig. 4:**
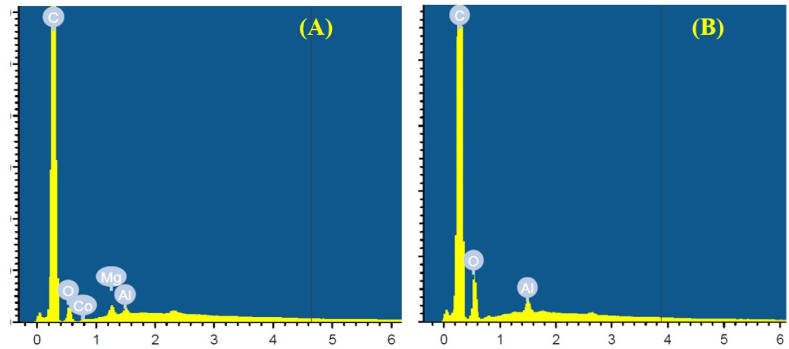
The x-ray dispersive spectroscopy study of multi-walled carbon nanotubes shows the elemental composition before chemical oxidation (A) and after functionalization (B).

The elemental composition of R-MWCNTs and O-MWCNTs was explored by EDS. Owing to the synthesis procedure, pristine R-MWCNTs contain a small fraction of catalyst and small oxygen content, (Fig. 4 A). The oxygen content increases in the functionalized O-MWCNTs (Fig. 4 B) due to oxidation, which reveals the presence of functional groups [30].

#### 3.2.3 Raman spectroscopy

In nanoscience, Raman spectroscopy shines as a non-destructive way to unveil a material's fingerprint and witness its evolution in real-time. This makes it invaluable for identifying types of nanomaterials, detecting defects, monitoring modifications, and studying their interactions with other molecules, all crucial for optimizing nanomaterials for diverse applications.

Raman spectroscopy was employed to characterize the defect structures of pristine and chemically treated MWCNTs in the wavenumber range of 500–3200 cm⁻^1^. Three characteristic peaks appeared at the D band at 1340 cm⁻^1^ (associated with disordered sp³-hybridized carbon atoms within the MWCNT structure), the G band at 1580 cm⁻^1^ (characteristic of the graphitic sp^2^-hybridized carbon atoms in MWCNTs), and the G′ band at 2700 cm⁻^1^ (the second-order overtone of the D band, indicating the presence of defects and providing information on the three-dimensional order of the MWCNTs).

**Fig. 5:**
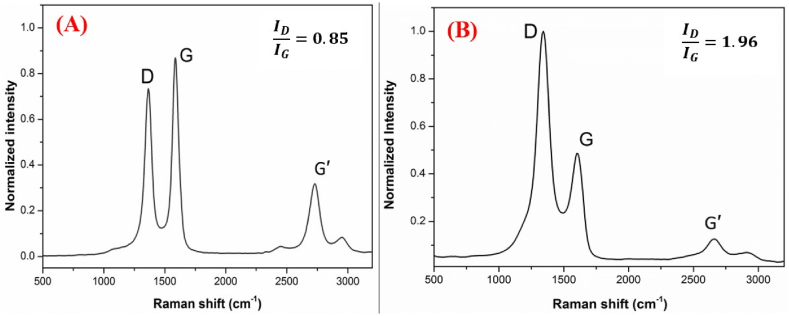
Raman spectra of pristine (A), and functionalized multi-walled carbon nanotubes (B), showing the I_D_/I_G_ ratio.

The analysis revealed an increase in D-band intensity (Fig. 5A and B) and a corresponding decrease in G-band intensity after chemical treatment. This translates to an increased ID/IG ratio (from 0.85 to 1.96), signifying a higher concentration of defects introduced into the MWCNT walls upon treatment. These findings align with the observations made through SEM [35,36]. Furthermore, the intensity variations in the G′ band support these observations. A stronger G′ band in pristine MWCNTs suggests a more ordered structure with fewer defects. This aligns with the concept that Raman spectroscopy can be used to quantify the degree of disorder associated with amorphous carbon deposits on MWCNT surfaces. The removal of these disordered regions through chemical treatment improves the overall crystallinity of the MWCNTs, further supporting the characterization of these surface defects.

#### 3.2.4 XRD characterization

**Fig. 6:**
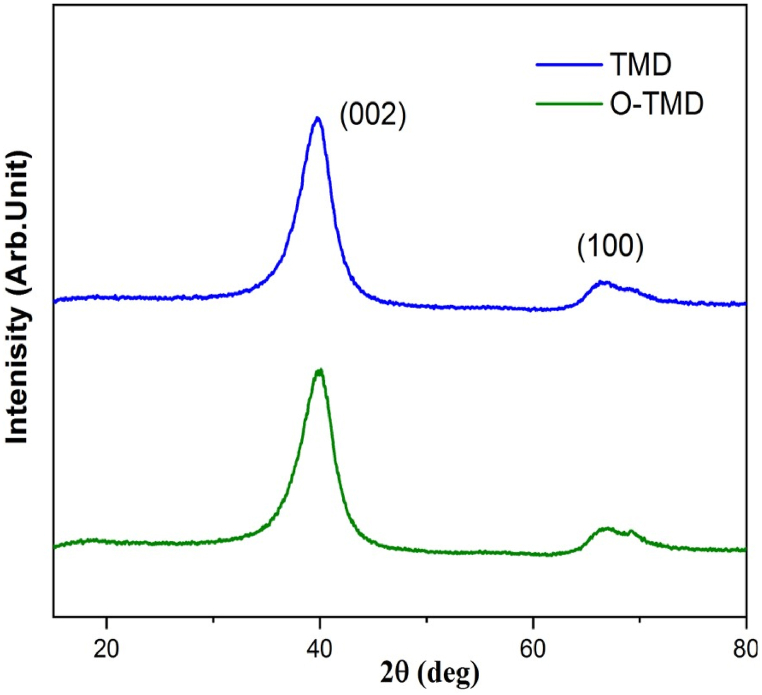
The pattern of x-ray diffraction for pristine, and functionalized multi-walled carbon nanotubes.

The pattern of XRD for pristine and functionalized MWCNTs is shown in Figure (6). Miller indices designate the two largest graphite-like Bragg peaks. The interlayer distances between the graphene planes are related to the (002) diffraction peak. On the other hand, the peak at 100 is typical of the two-dimensional in-plane symmetry that correlates with the graphene layers. The position and width of the (002) peak depend on the structural arrangement of the material. The R-MWCNTs sample showed angles of 39.65° and 40.2° for the pristine and oxidized samples, respectively. Lower diameter tubes have high curvature and correspondingly high strain, which leads to higher *d*-spacing. It also demonstrates that interlayer *d*-spacing between concentric tubes decreases as the diameter increases [14,35,36].

### 3.3 Optimization of the MB removal using SLD

To gain a reliable understanding of how *Trichoderma* sp. and MWCNTs collaborate and to pinpoint the best combination for achieving desired results, we used the SLD mixture. This approach enables efficient and empirically estimating the intricate relationship between these influencing factors and the response variables.

The SLD approach is novel in the context of MB removal using *Trichoderma*-MWCNTs composite and can potentially reveal new insights into the interactions between the composite and the dye. By varying the concentrations of *Trichoderma* mate and MWCNTs, we aim to identify the optimal conditions for maximum MB removal.

The SLDs are a mixture of protocols in which the design points are arranged in a uniform way (or lattice). The SLD is an experimental design method used in the field of quality engineering and optimization. The design involves creating a simplex, which is a combination of factor settings added to the interior of the design space, including the center of the simplex that represents the reference point or the current operating point. The simplex is then expanded or contracted by systematically varying the factor settings in a geometric pattern to explore the design space. The SLD is often used when the number of factors is relatively small, and the response surface is expected to be relatively smooth and continuous. It is particularly useful in situations where it is not feasible or practical to test all possible combinations of factor settings, as it allows for more efficient exploration of the design space [[23], [24], [25]].

To find out the optimum formula ratio of both hyphal mate of *Trichoderma* sp., and MWCNTs on MB removal, an advanced modeling procedure was applied. The experimental design of the SLD mixture was used for the formulation and optimization of the best combination of hyphal mate of *Trichoderma* sp., and MWCNTs. Table (1) displayed the matrix of the SLD mixture, the concentration of the two factors, and the corresponding actual and predicted data.

Data demonstrated obvious variance in MB removal depending on the combination nature of each run, ranging from 63.50 ± 0.29 to 95.78 ± 1.20 %, the latter was obtained at the center point (middle concentration). Based on the various formula observations, the 1: 1 mixture (formulation numbers 1 and 6) showed the highest removal of MB, with an average of 94.89 %. However, all formulas showed various degrees of MB removal.

The composite of *Trichoderma* sp. and MWCNTs has the potential to exhibit enhanced MB removal compared to *Trichoderma* sp. alone. This is due to the high surface area and unique adsorption properties of MWCNTs, and the strong enzymatic activities and metabolic pathways of Trichoderma spp [9,10,16].

#### 3.3.1 ANOVA and regression analysis

The obtained data from the SLD mixture were further analyzed using ANOVA (Table 2) to detect the adequacy of the design for modeling and predicting the optimum formulation conditions. The model was evaluated based on several measurements, including F-value, P-value, and lack of fit tests, together with R^2^, adjusted R^2^, and predicted R^2^.

**Table 2:** Analysis of variance of MB removal rate for component proportions (*Trichoderma* sp. mate and multi-walled carbon nanotubes) based on simplex-lattice mixture design.

Source	Degrees of freedom	Seq SS	Adj MS	F-value	P-value
Model regression	2	1221.47	610.73	245.06	0.000∗
Linear	1	64.58	64.58	25.91	0.001∗
Quadratic	1	1156.89	1156.9	464.21	0.000∗
Hyphal mate (g/L) × MWCNYs (g/L)	1	1156.89	1156.9	464.21	0.000∗
Residual Error	7	17.45	2.49		
Lack-of-Fit	2	8.83	4.41	2.56	0.172
Pure Error	5	8.62	1.72		
Total	9	1238.92			
Model mathematics					
Multiple correlation coefficient (R^2^)			98.59		
The adjusted R^2^			98.19		
The predicted R^2^			97.40		
Predicted residual sum of square (PRESS)			32.18		

The model F-value (245.06) implies the model is significant. Also, the P value of the model was less than 0.050, indicating the model is significant. All model terms, including the linear, and quadratic, showed the same significant trend. Furthermore, R^2^, adjusted R^2^, and predicted R^2^ showed high values, being 98.59, 98.19, and 97.40 %, respectively. The PRESS recorded a relatively small value of 32.18. On the other side, the lack of fit F-value (2.56) showed insignificant relative to the pure error. The P value of 0.172 means the insignificant lack-of-fit. The ANOVA of the regression model indicates that Fisher's (F*-*value = 245.06) revealed that the model is significant, which is an indication of the goodness of fit of the model [8].

In the regression model, the R^2^ value above 0.9 suggests a strong correlation between the independent and dependent variables [7]. In this connection, the current R^2^ value indicated that 98.59 % of variations in MB removal can be described by the SLD mixture model. However, a high R^2^ value does not always reflect a good regression model, and the adjusted R^2^ must also be considered comparatively high [16]. The adjusted R^2^ of 98.19 % indicates a robust fit between the model and the experimental data within the design space explored. This suggests strong agreement between predicted and observed values.

Predicted R^2^ serves as a statistical guide to assess how well a model predicts response values within the specific range of your experimental variables. It indicates the proportion of variance explained by the model in this limited data space [8]. The current R^2^ value prediction stands at 97.40 %, signifying the strong significance of the model in predicting methylene blue removal. Furthermore, this predicted R^2^ value aligns closely with both the R^2^ and adjusted R^2^ values obtained from experimental data, demonstrating a high level of agreement between observed and predicted values for MB removal.

The low value of 32.18 for PRESS in the model suggests a strong correlation between the real, and predicted values of MB removal. A smaller PRESS value signifies better predictive ability. Additionally, the predicted R^2^ calculated from PRESS provides a more user-friendly interpretation of the model's fit compared to PRESS itself. Notably, both the low PRESS and high predicted R^2^ suggest that the model generalizes well beyond the data used for its estimation, reducing the risk of overfitting [37].

The combined evidence from the lack-of-fit test (insignificant), high R^2^, adjusted R^2^, predicted R^2^, and F-value, coupled with the low PRESS value, strongly suggests the high accuracy and reliability of the model for predicting MB removal. Additionally, the highly significant P-values of the model terms indicate their meaningful contribution to the process. These findings collectively support the model's adequacy in representing MB removal by the proposed composite.

#### 3.3.2 Error analysis

The residual analysis is the alterations between the actual and predicted values, representing the portion of the validation data not explained by the SLD model. By examining the residuals, the potential impact of the mixture on the MB removal that ensures compliance with underlying data analysis assumptions could be evaluated [38].

**Fig. 7:**
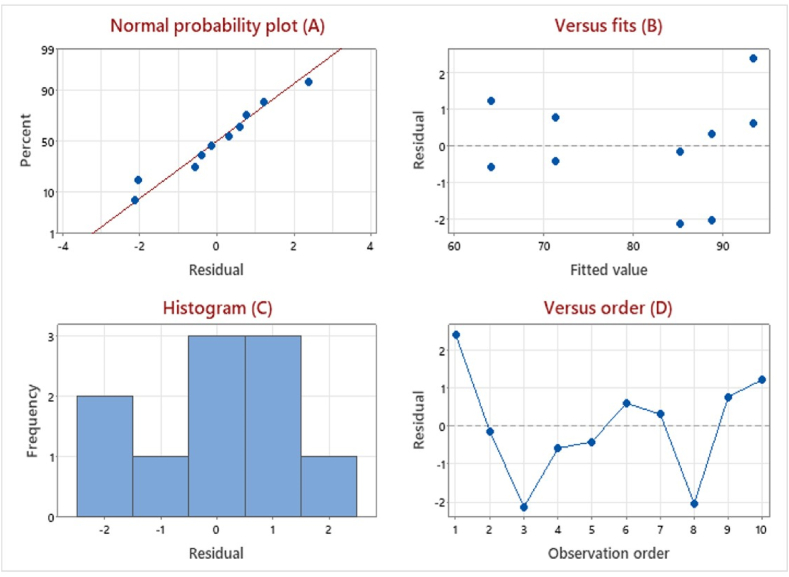
Residual plots for methylene blue removal rate by *Trichoderma* sp. mate and multi-walled carbon nanotubes composite.

Four key checks in Fig. 7 support the suitability of the chosen model for predicting MB removal. The normal probability plot (Fig. 7 A) follows a straight line, confirming the normality of residuals and the lack of outliers. Random distribution and constant variance of residuals around zero in the residuals vs. fit plot (Fig. 7 B) further solidify normality. Additionally, the symmetrical histogram without skewness or outliers in Fig. 7C reinforces this assumption. Finally, the random scatter around the centerline in the residuals vs. run order plot (Fig. 7 D) confirms residual independence, another key requirement [38]. Collectively, these analyses provide strong evidence that the model adheres to common assumptions, bolstering the validity of its predictions for MB removal. Accordingly, the SLD mixture is suitable for modeling the removal of MB by the proposed composite.

#### 3.3.3 Validation of the SLD model

Performing cross-validation offers a more comprehensive assessment of the model's reliability and predictive power. The optimal conditions for the maximal response by the hyphal mate-MWCNTs composite were predicted based on the model of the SLD mixture. The function of composite desirability (DF) was applied as a criterion for the selection of the best removal conditions of MB. Before experimental validation, the DF value, in the range of 0 (undesirable) to 1 (desirable), is often calculated mathematically to guide the optimization process. It serves as a quantitative indicator of the model's ability to predict optimal operating conditions that satisfy all desired criteria simultaneously [39].

**Fig. 8:**
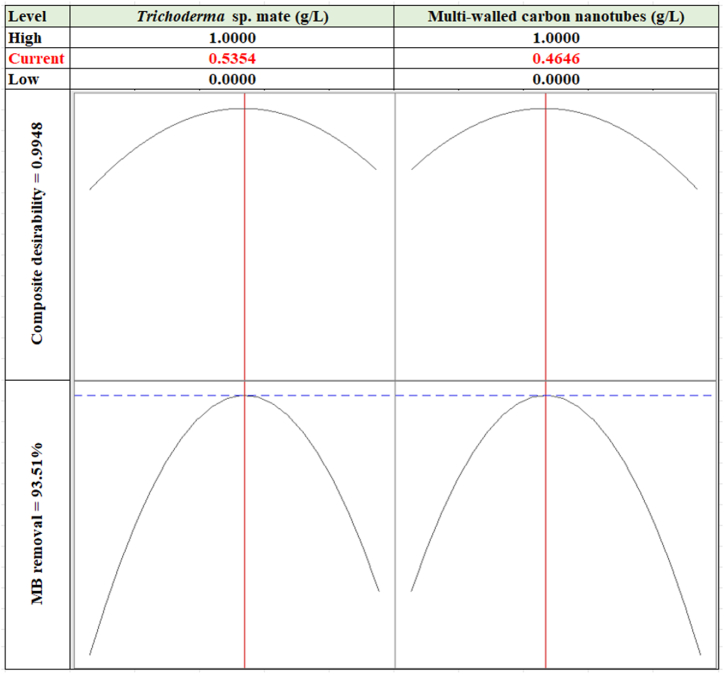
The optimization plot displays the optimum predicted values for the maximum methylene blue removal rate by *Trichoderma* sp. mate and multi-walled carbon nanotubes composite and the corresponding desirability function.

The optimization plot (Fig. 8) illustrates the DF and the estimated optimum values for maximum MB removal. As depicted, the optimum combined levels of hyphal mate and MWCNTs were found to be 0.5354, and 0.4646 g/L, respectively. The predicted MB removal was calculated to be 93.51 %. These calculated values were accompanied by a DF value of 0.9948.

These calculations were validated under laboratory conditions in triplicate using the predicted conditions. The resulting data showed a great consistency between the predicted and the experimental value of MB removal, being 94.01 ± 1.33 %. The close agreement between experimental and predicted values observed in the verification trial strongly supports the model's and DF's effectiveness in identifying optimal conditions for maximum MB removal by the proposed composite. This successful validation adds confidence to the predicted optimum and underscores the reliability of the DF approach in this specific context.

### 3.4 Decision tree learning algorithm

No previous research handled on the use of SLD and DT learning algorithms to enhance MB removal through a combination of hyphal mate and MWCNTs. The DT analysis was performed to identify the best combination of factors that predict the optimal range for the composite material.

The array of simplex-lattice mixture experimental design data with its data was used as historical data (Table 1) to train and construct the DT. Various learning algorithms were tested to choose the best DT model. After several trials, the best settings for the highest model performance (Table 3) were found to use the least squared error as a node splitting, and 10-fold cross-validation, applying two cases for the minimal number to split an internal node and one case for the minimal number allowed for a terminal node. Upon performing the learning process under such situations, the best DT that predicts MB removal by both predictors was obtained.

**Table 3:** Model and parameters of supervised decision tree learning algorithm for methylene blue removal rate by the proposed composite (*Trichoderma* sp. mate and multi-walled carbon nanotubes).

Model summary		
Number of predictors	2	
Significant predictors	2	
Terminal nodes number	5	
Minimum terminal node size	2	
Node splitting	Least squared error	
Validation	10-fold cross-validation	

**Model statistics**		
**Parameter**	**Training**	**Test**
R-squared	99.30 %	97.22 %

Root mean squared error (RMSE)	0.9284	1.8569
Mean squared error (MSE)	0.8620	3.4480
Mean absolute deviation (MAD)	0.9090	1.8180
Mean absolute percent error (MAPE)	0.0114	0.0227

DT model demonstrated high predictive accuracy, as evidenced by its strong R-squared value and low error metrics; RMSE, MSE, MAD, MAPE (Table 3). The two key parameters evaluated in the DT were found to be significant predictors of MB removal. These results suggest that the DT can effectively optimize MB removal by identifying the optimal parameter settings.

#### 3.4.1 Selection of the optimum DT model

The DT works by starting with a single root node that represents the entire dataset. During prediction, the tree from this root is navigated further. At each internal node, there is a decision question based on an input variable. By answering this question (Yes/No) or following a specific range, a decision is made to move down a specific branch of the tree. This process continues until reaching a leaf node, which provides the final prediction or classification for that data point. Essentially, DTs conduct a series of if-then-else rules based on the input variables. These rules progressively divide the population into smaller and more similar groups (homogeneous sets) until reaching a final classification. The splitting criteria at each node are chosen to maximize the difference between the resulting groups.

**Fig. 9:**
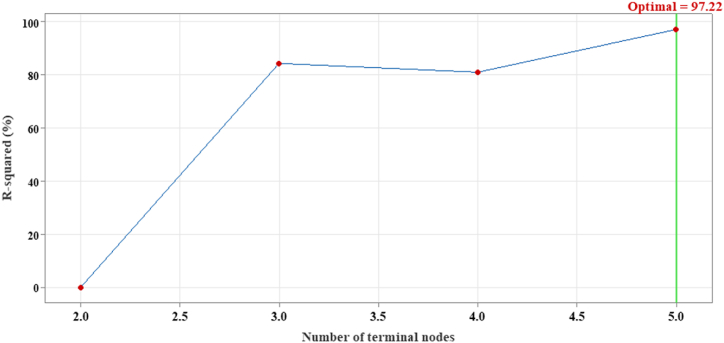
The generated terminal nodes versus R^2^ for the testing process by a DT for MB removal by the proposed composite of *Trichoderma* sp. mate and multi-walled carbon nanotubes.

To choose the optimal DT, R^2^ values of the terminal nodes were plotted (Fig. 9). This allows identifying the smallest DT that achieved the highest R^2^ value, indicating the best balance between model complexity and fit. Five terminal nodes representing 5 trees were generated. The maximized R^2^ values for the optimal regression tree were 99.30 % (training), and 97.22 % (testing).

#### 3.4.2 Relative importance of the variables

**Fig. 10:**
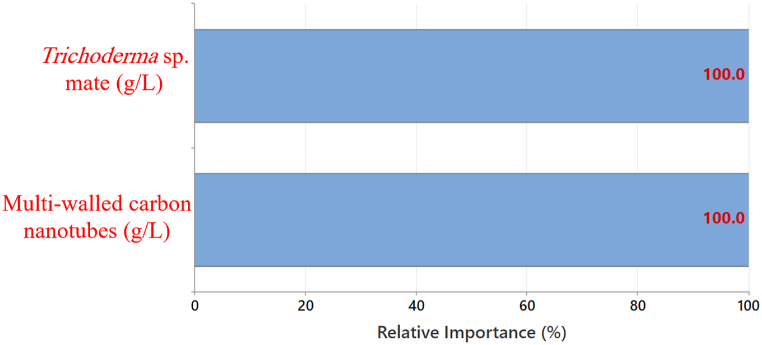
The relative variable importance of the two tested predictors on the methylene blue removal rate by the composite inferred by the decision tree.

In DTs, relative importance reflects how much a predictor variable contributes to splitting the data for better predictions. The most important variable is identified as the one with the highest improvement score (considered 100 % critical), in our case (Fig. 10) it was the hyphal mate. Then the other variables were ranked based on their relative improvement compared to the leading variable [27].

Although the hyphal mate was the leading (top) predictor, the contribution was 100 % in the MB removal rate by the composite. However, the other variable was also standardized as the top predictor, reflecting the importance of both predictors for the MB removal process. Therefore, to ensure accurate MB removal, close monitoring and control of both variables are crucial. This finding aligns with our initial hypothesis, as the DT effectively identified these hidden relationships between the tested parameters. However, this importance was, also, in line with that of the SLD.

#### 3.4.3 Constructing the optimal DT diagram

**Fig. 11:**
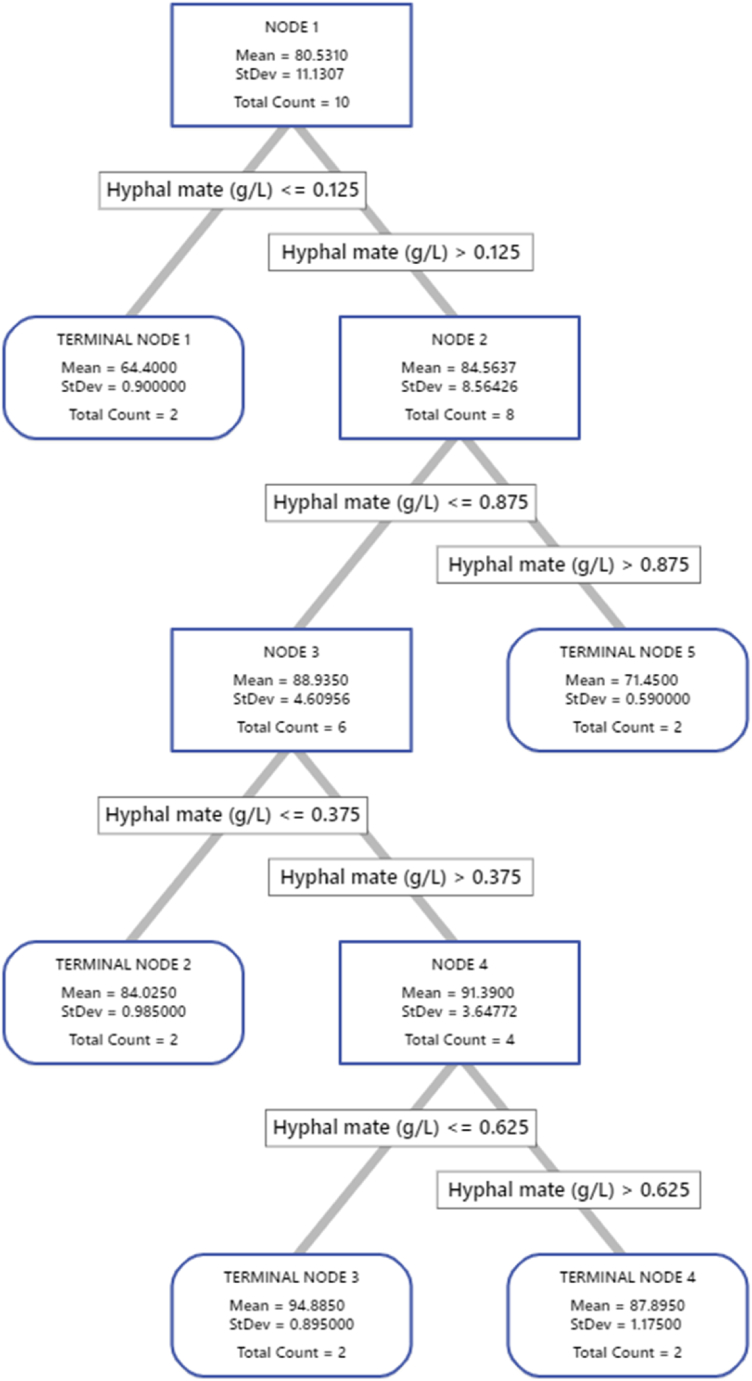
The decision tree diagram illustrates the methylene blue removal process based on 10 data cases. The squares within the diagram represent the data points used for node selection.

The DT diagram was depicted using 10 data sets (Fig. 11). The DT started with 10 cases (DT root) and split based on the level of hyphal mate into two branches, the first is at hyphal mate ≤ 0.125 g/L) and ends with terminal node 1, using only 2 cases. The second branch (hyphal mate >0.125 g/L) leads to node 2 with 6 cases. The latter was further split into terminal node 5 (hyphal mate >0.875 g/L) with 2 cases, and node 3 (hyphal mate ≤ 0.875 g/L) with 6 cases. The node 3 lead to the node 4, which ends with the third and fourth terminal nodes. Among the 5 terminal nodes, terminal node 3 (0.375 < hyphal mate (g/L) ≤ 0.625) was chosen as the best terminal node of the DT, achieving 84.89 % of MB removal, using 2 cases and further recording the low standard deviation (0.895) and maximum R^2^ values for training and testing. The other terminal nodes did not show further advances or were probably skewed.

#### 3.4.4 Error analysis of the DT model

**Fig. 12:**
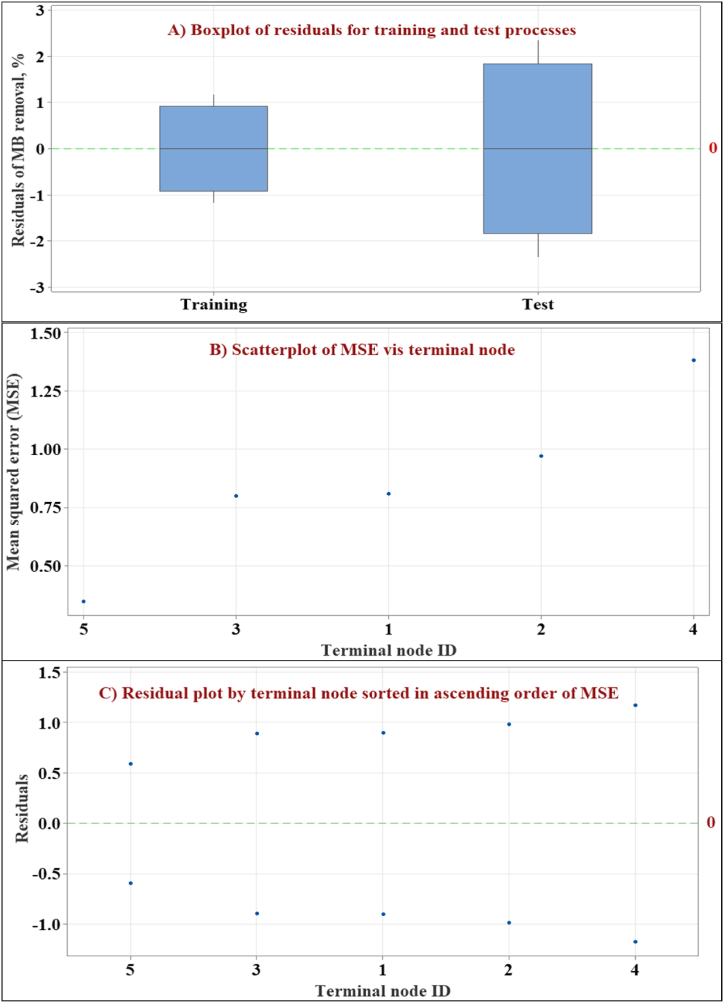
Error analysis of the constructed decision tree, showing the boxplot of residuals for training and test processes (A), scatterplot of mean squared error vis terminal node (B), and residual plot by terminal node sorted in ascending order of mean squared error (C).

The error analysis of the constructed DT model reveals insights into its performance and potential limitations of the MB removal model. Fig. 12 presents a comprehensive analysis through three visualizations of error tests.

The boxplot of residuals for training and test processes (Fig. 12 A) highlights a wider interquartile range for the residuals in the training process compared to the test process. This observation suggests potential low overfitting of the model to the training data. Overfitting occurs when a model prioritizes memorizing specific patterns within the training data, potentially leading to decreased performance on unseen data. The current SLD restricts the useable range of both predictors to a total of 100 %, limiting the model's application to this specific range. This confinement might mitigate concerns about overfitting. Additionally, the high R-squared values for both training (99.30 %) and testing (97.22 %) suggest a good overall fit of the model.

The scatterplot (Fig. 12 B) depicts the relationship between mean squared error and terminal node ID. The absence of a clear trend indicates that the MSE remains relatively consistent across the various terminal nodes within the decision tree. This suggests that the model's prediction errors are not concentrated in specific regions of the decision tree structure. This is a good sign, indicating that the model is not making significantly worse predictions in any region of the decision tree. Finally, the residual plot by terminal node is sorted in ascending order of mean squared error (Fig. 12C), which reinforces the notion of consistent MSE across the decision tree. The absence of clear patterns in the residual plot aligns with the scatterplot's observation of consistent mean squared error across terminal nodes [26,27]. This suggests good model performance within the SLD space, particularly in terms of prediction precision.

#### 3.4.5 Validation of DT models

The efficacy of the DT model was evaluated and compared to the model SLD for predicting MB removal in a system utilizing *Trichoderma* sp. hyphal mate and functionalized MWCNTs. The expected MB removal values from both models were compared with the actual measured values (Table 4). The results indicate that both models achieved a reasonable degree of prediction accuracy. The SLD model predicted a removal value of 93.51 %, with a corresponding actual value of 94.01 ± 1.33. Similarly, the DT model predicted removal efficiencies between 64.40 and 94.89 %, with actual values observed between 65.11 % (test point No. 1) and 95.18 % (test point No. 3). Notably, both models worked best at the level of both predictors, confirming the consistency of both models.

**Table 4:** The expected level of *Trichoderma* sp. mate and multi-walled carbon nanotubes based on simplex design and decision tree models and the predicted and actual MB removal values.

Examined point		Value of predictor (g/L)		Methylene blue removal, %		Terminal node ID
		Hyphal mate	MWCNTs	Predicted	Actual	
Simplex design		0.535	0.465	93.51	94.01 ± 1.33	
Decision tree	1	0.0	1.0	64.40	65.11 ± 4.32	1
	2	0.1	0.9	64.40	67.34 ± 1.85	1
	3	0.3	0.7	84.03	85.45 ± 4.01	2
	**4**	**0.5**	**0.5**	**94.89**	**95.18 ± 3.13**	**3**
	5	0.7	0.3	87.90	89.83 ± 2.18	4
	6	0.9	0.1	71.45	72.99 ± 6.05	5
	7	1.0	0.0	71.45	73.13 ± 2.55	5

Interestingly, our work surpasses existing methods. Compared to functionalized carbon nanotubes alone [40], our composite material with *Trichoderma* sp. achieves a significantly higher MB removal efficiency. Additionally, while activated carbon derived from agricultural wastes [41] offers similar efficiency, its preparation is more complex. Our approach also outperforms magnetic biochar [42] by not only demonstrating superior efficiency but also providing a more sustainable solution due to *Trichoderma* sp.'s natural biodegradability. Furthermore, the findings demonstrate the potential of both SLD and DT models for predicting MB removal in this system. Both models exhibited comparable prediction accuracy, suggesting that either approach could be a suitable choice for initial estimations. Additionally, the variability observed in the actual MB removal values highlights the inherent biological variability associated with the system.

### 3.5 Investigation MS biosorption

#### 3.5.1 SEM investigation

**Fig. 13:**
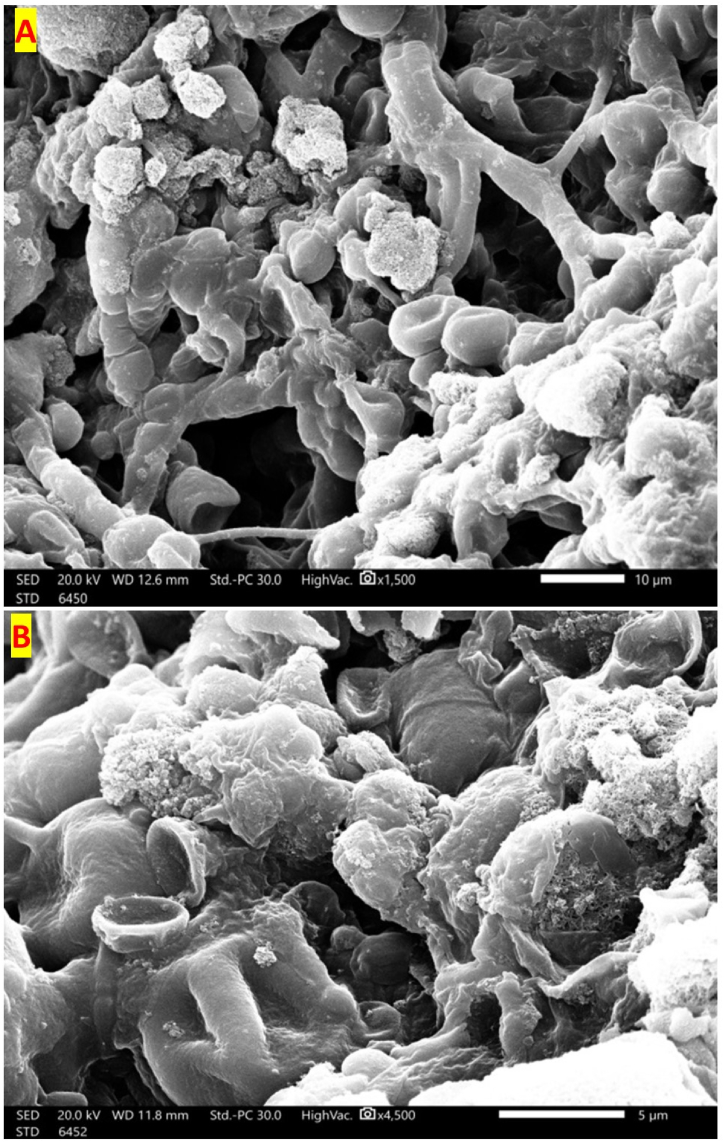
Scanning electron microscope images of the surface topology of the hybrid material of *Trichoderma* sp. mate and multi-walled carbon nanotubes: a comparison between the normal hybrid before (A) and after interacting with MB (B).

The best combination of hyphal mate-MWCNTs, obtained from the previous SLD was further investigated using SEM to elucidate the possible mode of action by which hyphal mate-MWCNTs absorbed MB. The application of the hyphal mate-MWCNTs mixture in the bioremediation of MB revealed distinct changes in the hyphal mate as detected by SEM image. The normal mixture led to MWCNTs adhering physically to the surface of the hyphae and becoming entangled within the hyphal network. Consequently, this resulted in potential changes to the surface topography of the hyphae (Fig. 13 A). The alteration in morphology indicated swelling and clustering of the hyphae, which created additional surface area and established a favorable environment for enhanced MB removal. On the other hand, the SEM image after interacting with MB (Fig. 13 B) further revealed a more concentrated distribution of dye on the hyphal surface compared to hyphae without MWCNTs.

The normal hybrid material also displayed a more distinct and organized surface morphology than the material that interacted with MB. The latter exhibited a rougher and less structured appearance, implying some degree of modification or deterioration due to the interaction with MB. The normal hybrid material demonstrated strong integration between the hyphal matrix and MWCNTs, forming a uniform and stable composite. In contrast, the material interacting with MB showed a lower level of integration, suggesting that the interaction with MB may have disrupted the bonds or interactions between the hyphal matrix and MWCNTs.

These findings suggest that the hybrid material has promise for the bioremediation of MB dye, given its ability to interact with and eliminate MB from water. However, the interaction with MB could influence the structural integrity and stability of the hybrid material, potentially impacting its effectiveness and longevity. Therefore, further research is needed to evaluate any changes in its physical and chemical properties resulting from the interaction. Thus, the next FT-IR analysis provides insights into the key chemical features, elucidating alterations stemming from the interactions between the hyphal mate and MWCNTs.

#### 3.5.2 FT-IR investigation

**Fig. 14:**
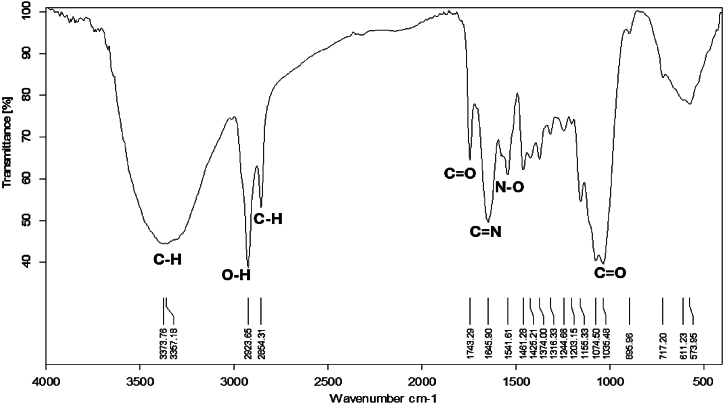
Fourier transform infrared spectroscopy of normal *Trichoderma* sp. mate and multi-walled carbon nanotubes interacting with water.

To identify the variation in functional groups in the hybrid material, FT-IR was used to compare it with the untreated mixture interacting with water. Both sets of spectra covered a wavenumber range of 4000-450 cm^−1^, where characteristic vibrations of various functional groups manifest. The FT-IR spectra of hyphal mate-MWCNTs interacting with water (Fig. 14), exhibited six major absorption peaks at 3373.76, 2923.65, 2854.31, 1645.90, 1035.48 and 611.23 cm^−1^ in the region of 450–4000 cm^−1^. The high absorption bands at 3373.76 correspond to the C–H stretching. The band at 1035.48 cm^−1^ corresponds to the C=O stretching [43,44].

**Fig. 15:**
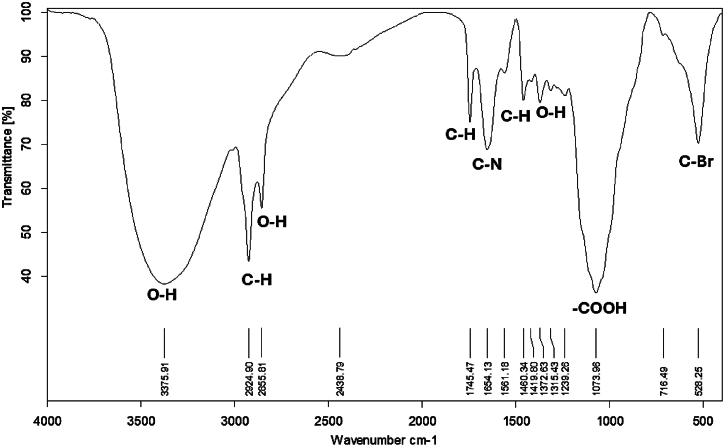
Fourier transform infrared spectroscopy of *Trichoderma* sp. mate and multi-walled carbon nanotubes interacting with MB.

The FT-IR spectrum of hyphal mate-MWCNTs interacting with MB (Fig. 15), exhibited five major absorption peaks at 3375.91, 2924.90, 1073.96, and 528.25 cm^−1^ in the region of 450–4000 cm^−1^. The high absorption bands were observed at 3375.91 –1073.96 cm^−1^. The peak at 3375.91 cm^−1^ can be assigned to the O-H stretch from carboxyl groups (O=C−OH and C−OH). The peak at 1073.96 cm^−1^ can be associated with the O−H stretching [45] from strongly hydrogen-bonded -COOH. Acidic oxidation of MWCNTs introduces numerous oxygen-containing functional groups onto their surface [11]. These groups not only enhance hydrophilicity, making MWCNTs more water-compatible but also create abundant adsorption sites. Consequently, the adsorption capacity of the MWCNTs increases, making them more effective in various applications, including ion exchange [46]. The FT-IR findings represented the presence of new functional groups on the surface of MWCNTs at 2438.79 and 528.25 cm^−1^. These functional groups are attributed to the conjugated biomolecules on the surface of CNTs acting as reducing and stabilizing agents.

Referring to the previous studies, Table (5) compares our approach with previous research on MB decolorization. Our composite of endophytic *Trichoderma* mate and MWCNTs achieved a higher removal rate than most prior studies. Matrices and microbial consortia also influenced efficiency. Most of the studies used eco-friendly microorganisms. These findings contribute to our understanding of microbial decolorization and suggest a potential for more effective bioremediation strategies.

**Table 5:** Methylene blue decolorization efficiency of various microorganisms in comparison to the current study.

Organism	Methylene Blue decolorization	Reference
Composite of endophytic *Trichoderma* Mate and Multi-Walled Carbon Nanotubes	94.01 %	Current study
A culture of *Ralstonia pickettii*, and *Trichoderma virid*e into a matrix of Sodium Alginate-Polyvinyl Alcohol-Bentonite	97.88 %	Nabilah et al. [47]
Mixed microfungal of *Aspergillus niger, A. flavus* and *A. fumigatus*	92.00 %	Karaghool [48]
*Penicillium* sp. FTM7	95.45 %	Permana and Awaluddin [49]
*Penicillium* sp. P1	85.00 %	Liu et al. [50]
*Fomitopsis pinicola*	*92.56 %*	Purnomo et al. [51]
*Alternaria* sp. D21	78.00 %	Toker et al. [52]
*Gloeophyllum trabeum*	85.00 %	Purnomo et al. [53]
Mixed culture of *Daedalea dickinsii* and *Aspergillus oryzae*	64.77 %	Purnomo et al. [54]
Mixed culture of *Daedalea dickinsii* and *Ralstonia pickettii*	89.00 %	Nabilah et al. [55]

MWCNTs, known for their high surface area and adsorption capabilities, have been explored for dye removal, often requiring functionalization. Here, we present a novel approach that surpasses previous single-functional-group methods. Our composite material, combining MWCNTs with endophytic *Trichoderma* mate and optimized through SLD, and DT, achieves significantly higher MB reduction. This unique ternary nanocomposite, unreported for MB adsorption before, represents a groundbreaking development in wastewater treatment.

The findings of this study significantly contribute to the existing body of literature on bioremediation and advanced material applications in wastewater treatment. The integration of endophytic *Trichoderma* sp. with MWCNTs presents a novel composite material that leverages the high adsorption capacity of MWCNTs and the biological degradation capabilities of *Trichoderma* sp. This synergistic approach not only enhances MB removal efficiency but also offers a sustainable and green option to traditional methods [56,57].

This proposed method utilizes a combination of endophytic *Trichoderma* sp. and MWCNTs, demonstrating significant potential for environmental sustainability and scalability. Endophytic fungi, such as *Trichoderma* sp., are known for their biotechnological applications, including bioremediation, due to their ability to produce bioactive compounds [16]. The integration of MWCNTs further enhances the adsorption capacity and stability of the composite, making it a robust solution for dye removal. The environmental impact of this method is minimal, as it utilizes naturally occurring fungi and carbon-based materials, reducing the reliance on chemical treatments that can be harmful to ecosystems [58,59].

In terms of scalability, the production of endophytic fungi can be efficiently scaled up using bioreactors, which have been successfully employed in various industrial applications [60]. The cost-effectiveness of this method is also notable, as the raw materials are relatively inexpensive, and the process can be optimized to maximize efficiency. Regulatory considerations include ensuring the safe handling and disposal of MWCNTs, which can be managed through existing guidelines for nanomaterials. The economic benefits of this method are substantial, offering a low-cost, high-efficiency solution for wastewater treatment, particularly in resource-limited settings [59,61]. The proposed method offers a sustainable, scalable, and economically feasible solution to address global water pollution challenges.

Furthermore, SLD and DT learning algorithms represent pioneering methodological advancement. These techniques enable a comprehensive exploration of variable interactions and provide robust predictive models, which are crucial for scaling up the process for industrial applications [26,27]. The study's innovative approach highlights the potential for practical application in resource-limited settings, effectively addressing global water pollution challenges.

While highly effective under controlled conditions, limitations exist. Real-world applicability needs evaluation in complex wastewater with varying contaminants. The efficiency at different MB concentrations and the long-term stability/reusability of the composite require further study. In the future, further research should also examine the economic viability and environmental consequences of large-scale usage. Addressing these aspects is expected to solidify the practical value of this innovative and eco-friendly solution for water treatment.

## 4 Conclusion

This study presents a novel and highly efficient (95.78 % removal) bioremediation approach for removing MB from water. The current composite material was optimized using SLD and DT learning algorithms, offering a promising solution for tackling MB pollution. MB is a common dye pollutant known to be harmful to aquatic life, and effective removal methods are crucial for environmental protection. The unique hybrid structure observed through SEM and the formation of novel functional groups confirmed by FT-IR suggests strong interactions between the endophytic fungi and MWCNTs, potentially aiding in MB adsorption. While the DT learning algorithm shows promise for optimizing MB removal, further research with larger datasets and incorporating real-world environmental factors are necessary for practical applications. Future investigations should also explore the composite's reusability, economic feasibility, and effectiveness in complex wastewater streams. Ultimately, this study not only advances knowledge in bioremediation and nanotechnology but also paves the way for developing sustainable and efficient strategies for MB removal from wastewater. Additionally, the findings shed light on the potential of endophytic fungi for enhancing the efficacy of nanomaterials in bioremediation applications. Kinetic and isotherms studies can offer deeper insights into the adsorption behavior, further guiding the development of this promising technology.

## CRediT authorship contribution statement

**Sahar E. Abo-Neima:** Writing – review & editing, Writing – original draft, Validation, Formal analysis, Data curation, Conceptualization. **Emad M. Elsehly:** Writing – review & editing, Writing – original draft, Visualization, Validation, Data curation, Conceptualization. **Fatimah O. Al-Otibi:** Writing – original draft, Resources, Funding acquisition, Formal analysis, Data curation. **Mohammed M. El-Metwally:** Writing – review & editing, Visualization, Supervision, Methodology, Data curation, Conceptualization. **Yosra A. Helmy:** Writing – original draft, Visualization, Validation, Formal analysis, Conceptualization. **Noha M. Eldadamony:** Writing – original draft, Visualization, Methodology, Formal analysis, Data curation. **WesamEldin I.A. Saber:** Writing – review & editing, Writing – original draft, Validation, Supervision, Methodology, Data curation, Conceptualization. **Adel A. El-Morsi:** Writing – review & editing, Visualization, Validation, Methodology, Formal analysis, Conceptualization.

## Data availability statement

All data generated or analyzed during this study are included in this published paper.

## Funding

This work is supported by Researchers Supporting Project number (RSP2024R114), 10.13039/501100002383King Saud University, Riyadh, Saudi Arabia.

## Declaration of competing interest

The authors declare that they have no known competing financial interests or personal relationships that could have appeared to influence the work reported in this paper.
